# Genetic diversity of *Plasmodium vivax* population in Anhui province of China

**DOI:** 10.1186/1475-2875-13-13

**Published:** 2014-01-08

**Authors:** Bo Huang, Shiguang Huang, Xin-zhuan Su, Hong Guo, Yucheng Xu, Fei Xu, Xuchu Hu, Yaming Yang, Shanqing Wang, Fangli Lu

**Affiliations:** 1 Department of Parasitology, Zhongshan School of Medicine, Sun Yat-sen University, Guangzhou 510080, Guangdong, China; 2 Key Laboratory of Tropical Disease Control (Sun Yat-sen University), Ministry of Education, Guangzhou 510080, Guangdong, China; 3 School of Medicine, Jinan University, Guangzhou 510632, China; 4 Laboratory of Malaria and Vector Research, National Institute of Allergy and Infectious Diseases, National Institutes of Health, Bethesda, MD 20892, USA; 5 State Key Laboratory of Cellular Stress Biology, School of Life Science, Xiamen University, Xiamen 361005, Fujian, China; 6 Public Laboratory of Hainan Medical University, 3 Xueyuan Road, Haikou 571199, Hainan, China; 7 Clinical Laboratory, Licang Town Hospital of Mengcheng County, Anhui province 233500, China; 8 Yunnan Institute of Parasitic Diseases, Puer 665000, Yunnan, China; 9 Hainan CDC, Haikou 570203, Hainan, China

**Keywords:** *Plasmodium vivax*, *pvmsp-1*, *pvmsp-3α*, *pvcsp*, Anhui, China

## Abstract

**Background:**

Although the numbers of malaria cases in China have been declining in recent years, outbreaks of *Plasmodium vivax* malaria were still being reported in rural areas south of the Yellow River. To better understand the transmission dynamics of *P. vivax* parasites in China, the extent of genetic diversity of *P. vivax* populations circulating in Bozhou of Anhui province of China were investigated using three polymorphic genetic markers: merozoite surface proteins 1 and 3α (*pvmsp-1* and *pvmsp-3α*) and circumsporozoite protein (*pvcsp*).

**Methods:**

Forty-five *P. vivax* clinical isolates from Bouzhou of Anhui province were collected from 2009 to 2010 and were analysed using PCR/RFLP or DNA sequencing.

**Results:**

Seven and six distinct allelic variants were identified using PCR/RFLP analysis of *pvmsp-3α* with *Hha*I and *Alu*I, respectively. DNA sequence analysis of *pvmsp-1* (variable block 5) revealed that there were Sal-I and recombinant types but not Belem type, and seven distinct allelic variants in *pvmsp-1* were detected, with recombinant subtype 2 (R2) being predominant (66.7%). All the isolates carried *pvcsp* with VK210 type but not VK247 or *P. vivax*-like types in the samples. Sequence analysis of *pvcsp* gene revealed 12 distinct allelic variants, with VK210-1 being predominant (41.5%).

**Conclusions:**

The present data indicate that there is some degree of genetic diversity among *P. vivax* populations in Anhui province of China. The genetic data obtained may assist in the surveillance of *P. vivax* infection in endemic areas or in tracking potential future disease outbreak.

## Background

Malaria kills more than a million people a year, and approximately 40% of the world’s populations live in malarious countries [1]. *Plasmodium vivax* is the most widespread species of human malaria parasites in the world and is endemic in many countries of Asia, Central and South America, the Middle East, and parts of Africa, with an estimated burden of 70–80 million cases annually [2,3]. In China, the malaria eradication campaign initiated in the 1990s, using vector control and drug treatment of febrile individuals had been highly successful. However, episodes of *P. vivax* malaria infection were still being reported, and *vivax* malaria re-emerged in many counties of central China such as provinces of Anhui, Shandong, Hubei, Henan, and Jiangsu in recent years [4]. Approximately 37% to 68% of malaria cases reported during 2003 to 2007 were from central China, where the predominant vector mosquito was *Anopheles sinensis*. Most importantly, the numbers of vivax malaria cases in Anhui province of China were the highest in the country during those years [5].

A countrywide malaria elimination policy was launched by the Ministry of Health of P.R. China in 2010, with the goal to eliminate malaria by 2015 in a majority of the regions with the exception of the border region in Yunnan province, and to completely eliminate malaria from China by 2020. To achieve this ambitious goal, studying population structure, genetic diversity, and transmission of *Plasmodium* parasites in endemic areas will provide important information for disease control and management, including baseline data essential for monitoring drug resistance and for predicting the origin and spread of parasite variants within and between populations, and the performance of malaria vaccines under development [6,7]. Several genetic markers including microsatellites and the genes encoding circumsporozoite protein (*csp*), merozoite surface protein 1 (*msp-1*), and merozoite surface proteins 3α and 3β (*msp-3α* and *msp-3β*), have been used to study *P. vivax* population diversity [8-11]. However, so far the population structures of *P. vivax* are less well studied and understood and only a limited number of studies on the population genetics of *P. vivax* isolates in China have been conducted [7]. In this study, the population diversity of clinical *P. vivax* isolates from Anhui province of central China was evaluated using the above three polymorphic markers, and the molecular diversity data may provide important information for vivax malaria surveillance and for tracking parasites in future outbreaks in this area.

## Methods

### Study area

**Figure 1:**
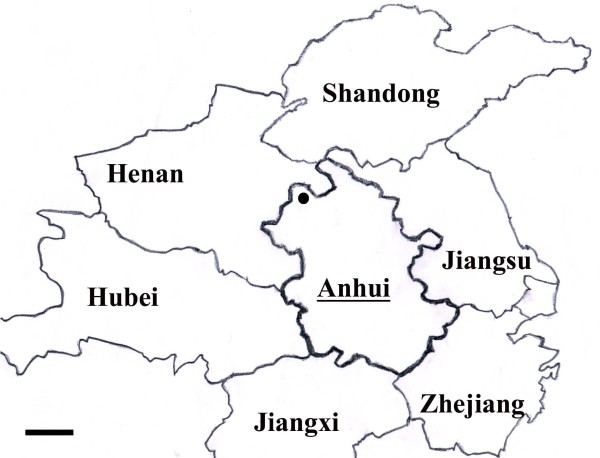
**Map of Anhui province in central China showing the location of Bozhou city where *P. vivax *isolates were collected.** Scale bar = 100 kilometer.

The study was conducted in Bozhou city of Anhui province, which borders six provinces: the Jiangsu province in the east, the Zhejiang province in the southeast, the Jiangxi province in the south, the Hubei province in the west, the Henan province in the northwest, and the Shandong province in the north (Figure 1), located in an area between latitudes 32°51′ and 35°05′, and longitudes 115°53′ and 116°49′. The population of this city was estimated to be 485,000 in 2010. The area has a distinct seasonal climate characterized by humid summer from June to September and dry winter from October to December, with temperature ranging from 14°C to 17°C and rainfall ranging from 500 mm to 750 mm per year [12]. Bozhou city was a vivax endemic area with seasonal transmission peaking from June to September. Climatic and ecological conditions of this area make the environment favorable for mosquito breeding, and the main malaria vector is *An. sinensis* and the secondary one is *Anopheles anthropophagus*.

### Patients and sample collection

This study was reviewed and approved by the Ethics Committee of Sun Yat-sen University, China (IRB 2009A0101). Blood samples were collected from patients with symptomatic *P. vivax* malaria admitted to the local hospitals for antimalarial drug treatment in the city from June 2009 to October 2010. Inclusion criteria were patients infected with *P. vivax,* but not other human malaria species, as confirmed by peripheral smear examination. Preparation and staining of the blood slides were done following the procedures of WHO outlined in basic malaria microscopy. From patients who had the fever-chill cycle and visited a designated hospital for malaria diagnosis and treatment in Bozhou area, forty-five *P. vivax*-infected patients were diagnosed by Giemsa-stained thin and thick blood smears, in which only two out of the 45 *P. vivax* infected patients had a travel history outside of Anhui province, and none of the patients had a travel history abroad. After informed consent from all adults or legal guardians of children, 1.0 ml of whole blood sample was collected in EDTA and stored at -20°C until DNA extraction.

### DNA extraction

*Plasmodium vivax* genomic DNA was extracted from 200 μl of each infected blood sample using DNA blood kit following the manufacturer’s instructions (TaKaRa, Japan) with minor modifications. The DNA was dissolved in TE buffer (10 mM Tris–HCl, pH 8.0, 0.1 M EDTA) and stored at -20°C until use. The quality of total DNA was analysed by running 5 μL of each DNA sample on a 1.0% agarose gel stained with ethidium bromide and visualized with UV illumination.

### PCR amplification of *pvmsp-3α*, *pvmsp-1*, and *pvcsp* genes

To amplify the *pvmsp-3α*, *pvmsp-1*, and *pvcsp* genes, a nested PCR amplification method was used following previously reported protocols with some minor modifications [10,13,14]. Oligonucleotide primers and cycling conditions are listed in Additional file 1. All amplification reactions were carried out in a total volume of 25 μL containing 17.0 μl of dH_2_O, 1.0 μl of each primer (10 pM), 1.5 μl of MgCl_2_ (25 mM), 0.5 μl of Taq polymerase (5 U/μl), 0.5 μl of dNTP mixture (10 mM each), and 2.5 μl of 10 × PCR buffer, following the manufacturer’s instructions (BBI, Canada). Primary amplification reactions were initiated with the addition of 1.0 μL of template genomic DNA prepared from the blood samples, and 1.0 μL of the primary reaction amplification was used as template in the secondary amplification reactions. The amplified PCR products were resolved on 1.0% agarose gel, and the sizes of the PCR products were determined using a 100 or 500 bp DNA ladder (NewProbe, China).

### PCR/RFLP analysis of *pvmsp-3α* gene

For RFLP analysis of *pvmsp-3α* gene, the PCR products were digested individually with restriction enzymes *Hha*I and/or *Alu*I in 20 μl reaction volumes at 37°C for 4 h as previously described [10]. Briefly, all digestion reactions were carried out in the presence of 10.0 μl PCR product, 7.0 μl of dH_2_O, 1.0 μl of enzymes *Hha*I or *Alu*I (5 U/μl), and 2.0 μl of buffer according to the manufacturer’s instructions (TaKaRa, Japan). After electrophoresis on 2.5% agarose gel, the enzyme-digested fragments were visualized under UV illumination. The sizes of the digested fragments were estimated using a 100 bp ladder of molecular weight markers. The results were recorded and analysed on a Gel Doc XR image analyzer using Quantity One software (Tanon, China).

### Sequence analysis of *pvmsp-1* and *pvcsp* genes

The nested PCR products of *pvmsp-1* and *pvcsp* genes were directly sequenced in both directions using an ABI PRISM3730 DNA sequencer by Sangon Biotech (Shanghai, China). Nucleotide and amino acid sequences were aligned and compared using CLUSTAL W of BioEdit 7.0 program with the following published sequences: VK210 (accession no. M28746) and VK247 (accession no. M28745) of *pvcsp* and Sal-I (accession no. M75674) and Belem (accession no. M60807) of *pvmsp-1*, respectively. Sequence relationship trees of the *pvmsp-1* and *pvcsp* genes from the Anhui isolates and published sequences from isolates of different geographic locations of Asia were constructed using neighbour-joining method implemented in MEGA 4 program [15]. Bootstrap proportions were used to assess the robustness of the tree with 1,000 bootstrap replications.

## Results

### PCR/RFLP analysis of the *pvmsp-3α* gene

**Figure 2:**
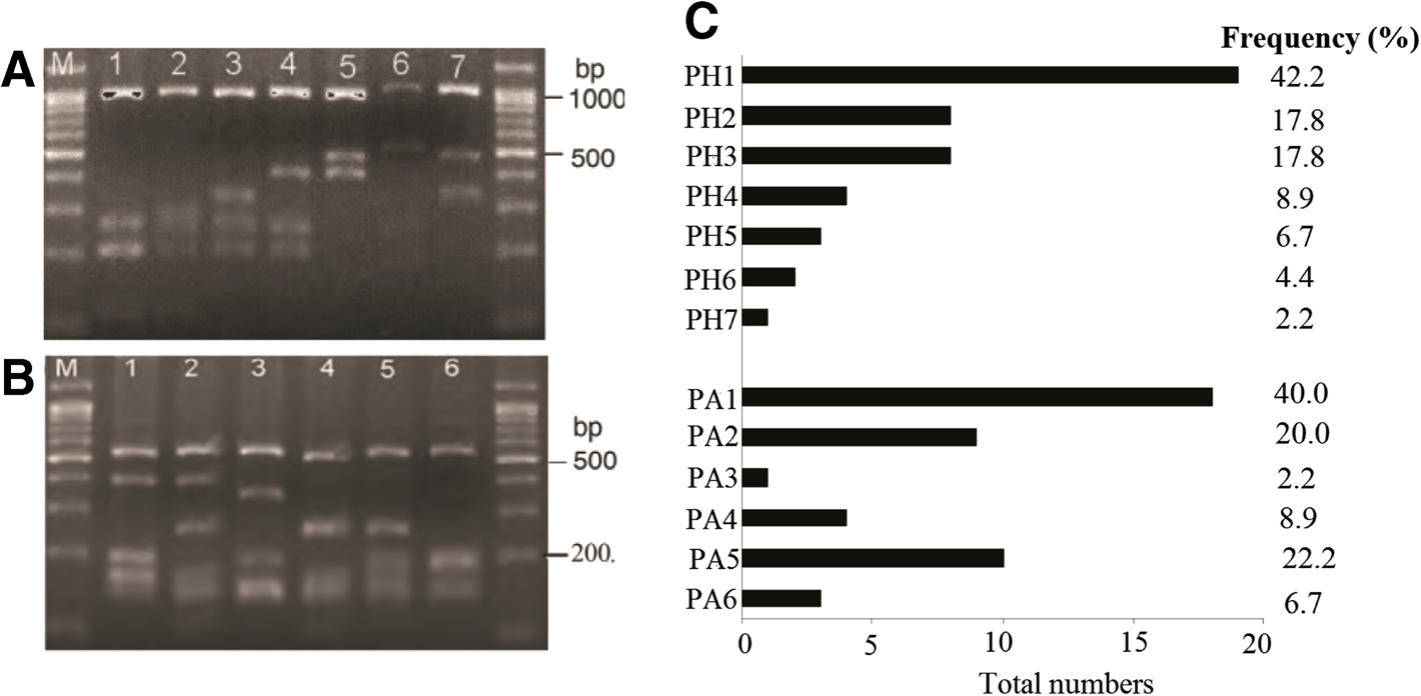
**PCR/RPLF genotypes and allelic frequencies of 45 *P. vivax *Anhui isolates based on *pvmsp-3α*. (A)** The amplification products of *pvmsp-3α* observed in Anhui *P. vivax* isolates were digested by *Hha*I, and seven variants were obtained (PH1 to PH7). **(B)** The amplification products of *pvmsp-3α* observed in Anhui *P. vivax* isolates were digested by *Alu*I, and six variants were obtained (PA1 to PA6). The lane with the molecular weight marker (100 bp ladder) is labeled as M. **(C)** The allelic frequencies of *pvmsp-3α* after *Hha*I *or Alu*I restriction from Anhui *P. vivax* isolates. **PH**: The allelic type of *pvmsp-3α* was based on the fragment sizes of *Hha*I-digested PCR products; **PA**: The allelic type of *pvmsp-3α* was based on the fragment sizes of *Alu*I -digested PCR products.

The *pvmsp-3α* gene was successfully amplified from all the 45 *P. vivax* isolates examined. All of the PCR products from *P. vivax* isolates were the same size of approximately 1.9 kb (type A); no type B (~1.5 kb) or type C (~1.1 kb) was detected. To further characterize variation in the gene, the PCR products were digested with restriction enzymes *Hha*I and *Alu*I, and 7 (PH1 to PH7) and 6 (PA1 to PA6) distinct variants were detected, respectively, based on the restriction patterns of the PCR products (Figures 2A, B). In the *Hha*I digestion, allelic variants PH1, PH2, and PH3 were the most common patterns, with frequencies of 42.2%, 17.8%, and 17.8%, respectively (Figures 2C), representing 77.8% of the total samples tested. Similarly, 82.2% of the *Alu*I digested samples were allele variants of PA1, PA2, and PA5, representing 40.0%, 20.0%, and 22.2%, respectively (Figures 2C).

### DNA sequence polymorphism and clustering of *pvmsp-1*

**Figure 3:**
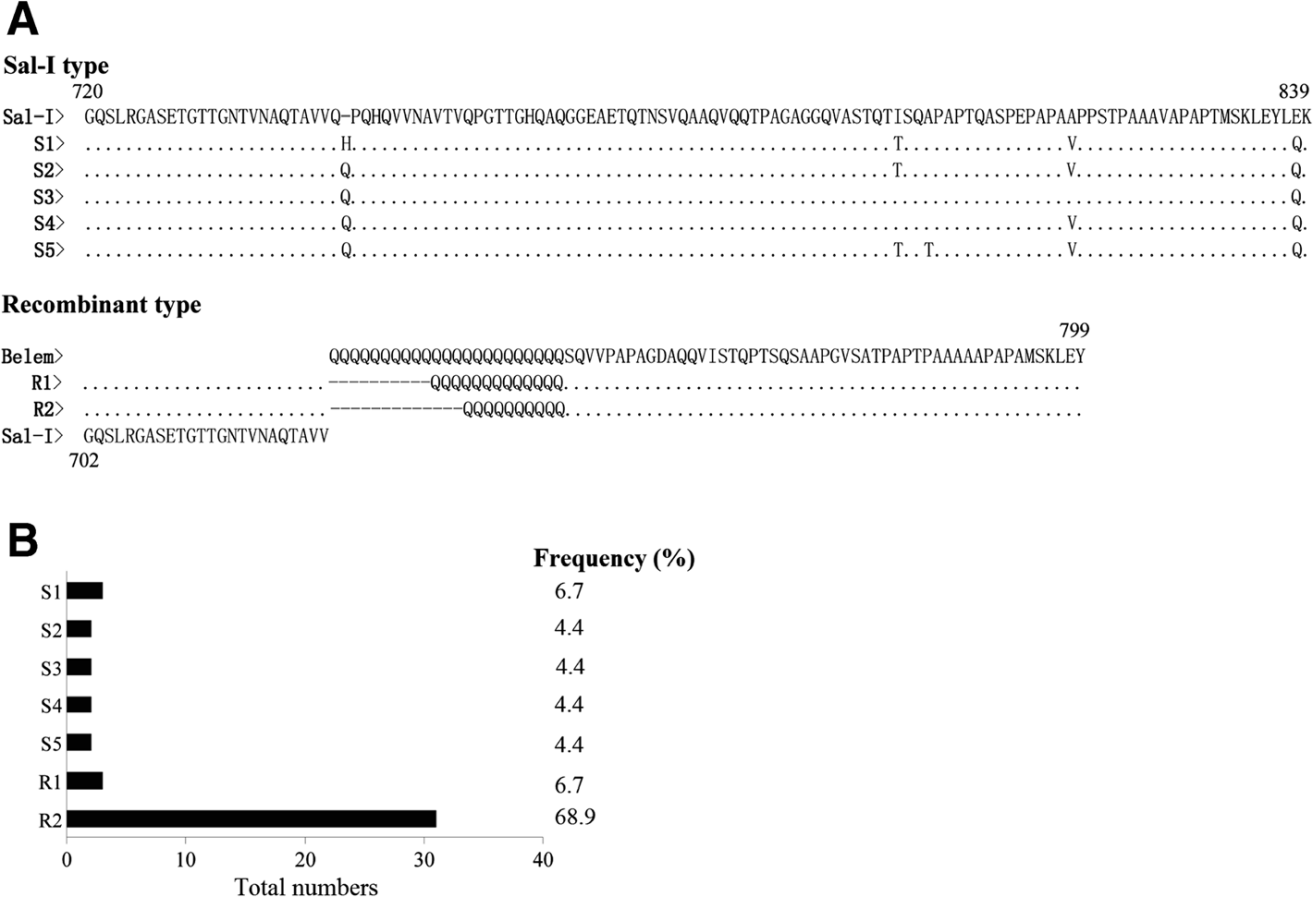
**Alignment of amino acid sequences of 7 *pvmsp-1 *distinct allelic variants and frequencies of *pvmsp-1 *allelic variants identified from 45 *P. vivax *Anhui isolates. (A)** Sequences were constructed to the published amino acid sequences of Belem (M60807) and Sal-I (M75674). Dots and dashes represent identical residues and deletions, respectively. Amino acid changes resulting from nucleotide substitutions are shown in bold. S: Sal-I type; R: Recombination type. **(B)** Allelic frequencies of 7 *pvmsp-1* allelic variants obtained from *P. vivax* Anhui isolates. S: Sal-I type; R: Recombination type of *pvmsp-1*.

All 45 isolates from Bozhou were successfully amplified for *pvmsp-1* gene. The size of PCR products varied from 420 to 520 bp for the isolates examined, with the 420 bp product being the most common fragment (80.2%). Sequence analyses of the PCR products showed that the isolates could be divided into two types: Sal-I (S) type (24.4%) or a recombination (R) type (75.6%) that was characterized by a combination of a Sal-I-like sequence at the 5′-end and a Belem-like sequence at the 3′-end, including a polymorphic poly Q segment [8,16]; no Belem type was found in these isolates. In total, seven distinct variants were found in the isolates, with five variant subtypes for the Sal-I type and two variant subtypes for the R-type, and with 13 and 10 poly Qs, respectively (Figure 3A). Of the seven sequence variants, R2-type was the most prevalent, accounting for 66.7% of all isolates examined. Sal-I type 5 variants were evenly distributed among the isolates, with frequencies of 6.7%, 4.4%, 4.4%, 4.4%, 4.4%, and 6.7% for Sal-I type S1, S2, S3, S4, S5, and R1 types, respectively (Figure 3B).

**Figure 4:**
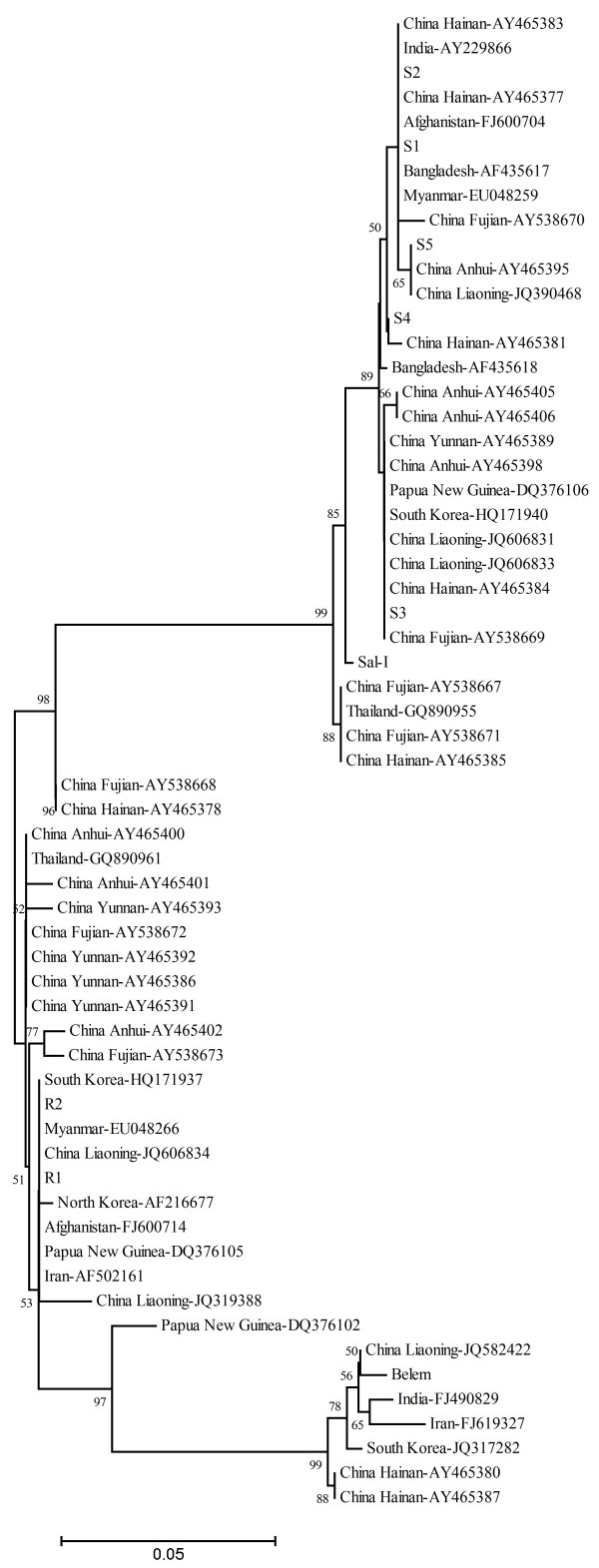
**Dendrogram of the *pvmsp-1 *gene based on the nucleotide acid sequences of 7 allelic variants from 45 *P. vivax *Anhui isolates and from 52 published sequences around the world in public databases.** The tree was constructed using neighbor-joining method implemented in MEGA4 program. The geographical origin of the 52 *pvmsp-1* published sequences is as follows: Anhui province (AY465395, AY 465398, AY465400, AY465401, AY465402, AY465405, and AY465406), Liaoning province (JQ319388, JQ582422, JQ390468, JQ606831, JQ606833, and JQ606834), Fujian province (AY538667, AY538668, AY538669, AY538670, AY538671, AY538672, and AY538673), Hainan province (AY465381, AY465377, AY465384, AY465378, AY465383, AY465385, AY465380, and AY465387), and Yunnan province (AY465389, AY465391, AY465392, AY465393, and AY465386) of China, Afghanistan (FJ600704 and FJ600714), Bangladesh (AF435617 and AF435618), India (AY229866 and FJ490829), Iran (FJ619327 and AF502161), Myanmar (EU048259 and EU048266), North Korea (AF216677), Papua New Guinea (DQ376102, DQ376105, and DQ376106), South Korea (HQ171940, JQ317282, and HQ171937), Thailand (GQ890955 and GQ890961), Belem (M60807), and Sal-I (M75674). The length of the line (bottom) is proportional to the genetic differences (%). Numbers on the branches indicate bootstrap proportions (1000 replicates). Only bootstrap values above 50% are displayed on the tree. S: Sal-I type; R: Recombination type.

A dendrogram of the *pvmsp-1* variable block 5 sequences was constructed using nucleotide sequences from the Anhui isolates and 57 published *pvmsp-1* sequences obtained from the isolates of different geographic locations of China (seven from Anhui province, seven from Fujian province, eight from Hainan province, six from Liaoning province, and five from Yunnan province of China) and other Asian countries (three from Afghanistan, two from Bangladesh, three from India, three from Iran, two from Myanmar, one from North Korea, three from South Korea, four from Papua New Guinea, three from Thailand, and two including the Sal-I strain and Belem strain from Brazil). This analysis grouped the *P. vivax* Anhui population into two major clusters (Sal-I type and R-type) with different subtypes in each group (Figure 4). The Sal-I type strains clustered with isolates from Hainan, Fujian, and Anhui of China as well as those from Afghanistan, Myanmar, Bangladesh, India, Thailand, and Papua New Guinea, whereas the R-type parasites clustered with parasites from Liaoning of China (northern China), North Korea, Iran, Afghanistan, Myanmar, and Papua New Guinea. Some of the *P. vivax* isolates examined in this study showed 100% identity with strains from other regions of China or even from different Asian countries, including Anhui province [AY465398 with S3 (Sal-I type)], Liaoning province [JQ606831 with S3, and JQ606834 with R2], Fujian province [AY538672 with R2], Hainan province [AY465377 with S2], Yunnan province [AY465391 with R2], South Korea [HQ171940 with S3, and HQ171937 with R2], North Korea [AF153032 with R2], India [AY229866 with S1], and Iran [AY502161 with R1]. Sal-I type S4 and S5 allele variants were new alleles identified in this study.

### DNA sequence polymorphism and clustering of *pvcsp*

**Figure 5:**
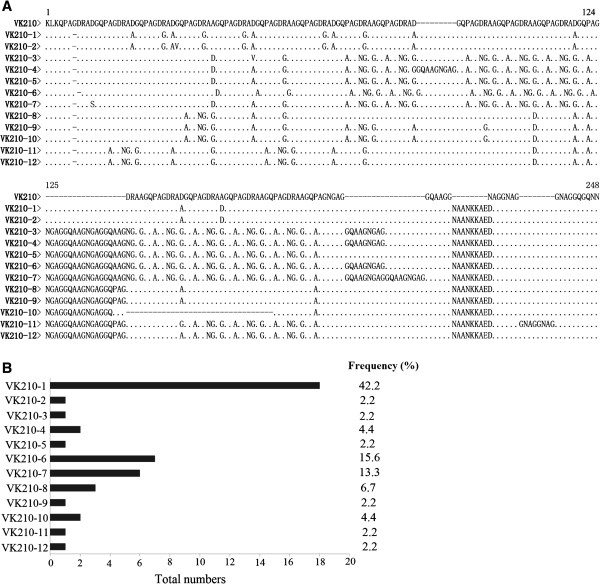
**Sequence alignments of amino acid sequences of 12 *pvcsp *distinct allelic variants and frequencies of *pvcsp *allelic variants identified from 45 *P. vivax *Anhui isolates. (A)** Sequences were aligned to the amino acid sequences of the reference strain VK210 (M28746). Dots and dashes represent identical residues and deletions, respectively. Amino acid changes resulting from nucleotide substitutions are shown in bold. **(B) ** Allelic frequencies of 12 *pvcsp* allelic variants obtained from *P. vivax* Anhui isolates.

To analyse polymorphisms in the *pvcsp* gene, the central repeat and its flanking regions of the gene from the samples examined were amplified and sequenced. All of the 45 isolates were successfully amplified for the *pvcsp* gene with PCR products having sizes ranging from 540 to 720 bp, with a 600 bp fragment being the predominant product among these isolates (58.5%). All of the DNA sequences belonged to VK210 type with 12 distinct variants; no VK247 and *P. vivax*-like types were detected within these isolates (Figure 5A) [17-19]. All variants started with the same pre-repeat sequence (KLKQP Region). The isolates displayed variations in the central peptide repeat motifs GDRA (A/D) GQPA with alternations of the repeating units, ranging from 7 to 17 repeats among these isolates. Of the 12 variants, the most prevalent sequence variant was VK210-1 subtype (42.2%, 19/45), followed by subtypes VK210-6 (15.6%, 7/45) and VK210-7 (13.3%, 6/45). The remaining nine variants of *pvcsp* gene were evenly distributed among the isolates (Figure 5B).

**Figure 6:**
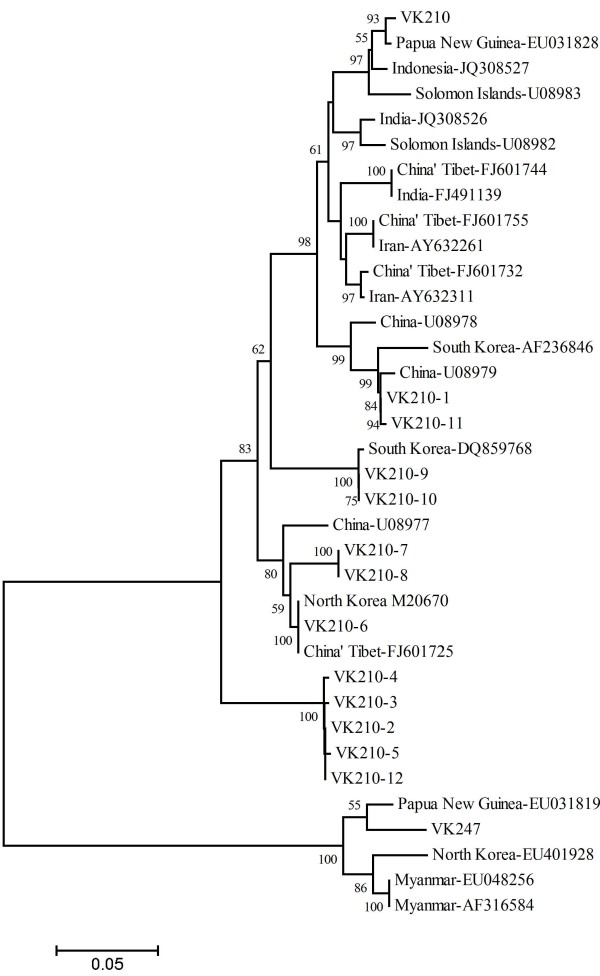
**Dendrogram of the *pvcsp *gene constructed (neighbor-joining method using by MEGA4 program) based on the nucleotide acid sequences from 12 allelic variants of 45 *P. vivax *Anhui isolates and from 22 published sequences around the world.** The geographical origin of the 22 *pvcsp* published sequences is as follows: China’s Tibet (FJ601725, FJ601755, FJ601744, and FJ601732), China (U08977, U08978, and U08979), India (JQ308526 and FJ491139), Indonesia (JQ308527), Iran (AY632261 and AY632311), Myanmar (EU048256 and AF316584), North Korea (M20670 and EU401928), Papua New Guinea (EU031819 and EU031828), the Solomon Islands (U08983 and U08982), South Korea (DQ859768 and AF236846), VK210 (M28746), and VK247 (M28745). The length of the line (bottom) is proportional to the genetic differences (%). Numbers on the branches indicate bootstrap proportions (1000 replicates). Only bootstrap values above 50% are displayed on the tree.

A dendrogram of the *pvcsp* sequences was constructed using neighbor-joining method based on the nucleotide sequences from Anhui isolates in this study and 22 published *pvcsp* sequences from the isolates of different geographic locations of Asia (seven from China, one from Indonesia, two from India, two from Iran, two from Myanmar, two from North Korea, two from Papua New Guinea, two from the Solomon Islands, and two from South Korea) (Figure 6). The sequences clustered into two distinct groups for the VK210 and VK247 type isolates. And the analysis clearly showed that all subtypes of *pvcsp* from Anhui isolates clustered with the VK210 type in the tree. Some of the *P. vivax* Anhui isolates in this study showed 100% identity with other strains from China and Asian countries previously published, such as China’s Tibet (FJ601725 with VK210-6), North Korea (M20670 with VK210-6), and South Korea (DQ859770 with VK210-10); whereas the remaining VK210 subtypes were new alleles identified in this study.

### Haplotypes from combined *pvmsp-3α*, *pvmsp-1*, and *pvcsp* allelic variants

By analysing variants of the three genetic markers (*pvmsp-3α*, *pvmsp-1*, and *pvcsp*) in combination, a total of 16 *P. vivax* haplotypes were identified in the 45 Anhui isolates with haplotype A of PH1 and PA1 of *pvmsp-3α*, R-2 of *pvmsp-1*, and VK210-1 of *pvcsp* being the most common haplotypes (37.8%), followed by haplotype D of PH2 and PA2 of *pvmsp-3α*, R-2 of *pvmsp-1*, and VK210-6 of *pvcsp* (Table 1). These haplotypes also contained the most common allelic variants of the individual markers. PH1 and PA1 of *pvmsp-3α*, R-2 of *pvmsp-1*, and VK210-1 of *pvcsp* were detected with high frequency in their corresponding genetic markers: PH1 and PA1 of *pvmsp-3α* (42.2% and 40.0%, respectively), R-2 of *pvmsp-1* (66.7%), and VK210-1 of *pvcsp* (42.2%).

**Table 1:** **Combined haplotypes and frequencies of *pvmsp-3α*, *pvmsp-1*, and *pvcsp *** **from 45 vivax isolates of Anhui province**

**Allelic variants**	**Genes**				**Number (%)**
	***pvmsp-3α***		***pvmsp-1***	***pvcsp***	
A	PH1	PA1	R-2	VK210-1	17 (37.8)
B	PH1	PA1	R-2	VK210-11	1 (2.2)
C	PH1	PA5	R-2	VK210-1	1 (2.2)
D	PH2	PA2	R-2	VK210-6	7 (15.6)
E	PH2	PA2	R-2	VK210-1	1 (2.2)
F	PH3	PA5	S-3	VK210-7	2 (4.4)
G	PH3	PA5	R-1	VK210-5	1 (2.2)
H	PH3	PA5	R-1	VK210-3	1 (2.2)
I	PH3	PA5	R-2	VK210-8	3 (6.6)
J	PH3	PA5	R-2	VK210-9	1 (2.2)
K	PH4	PA4	S-2	VK210-7	2 (4.4)
L	PH4	PA4	S-4	VK210-7	2 (4.4)
M	PH5	PA6	S-1	VK210-12	1 (2.2)
N	PH5	PA5	S-1	VK210-4	2 (4.4)
O	PH6	PA2	S-5	VK210-10	1 (2.2)
P	PH6	PA3	S-5	VK210-10	1 (2.2)
Q	PH7	PA5	R-1	VK210-2	1 (2.2)

**PH**: The allelic type of *pvmsp-3α* was based on the fragment sizes of *Hha*I-digested PCR products; **PA**: The allelic type of *pvmsp-3α* was based on the fragment sizes of *Alu*I -digested PCR products.

**S**: Sal-I type; **R**: Recombination type of *pvmsp-1*.

## Discussion

Malaria is one of the major parasitic diseases having a wide distribution in China with the prevalence gradually decreasing from south to north. Compared with southern (Hainan province) and southwestern (Yunnan province) regions, central China (including Anhui province) has a low endemicity of malaria, with *P. vivax* being the only species after the year 2000. Anhui province is the most seriously affected area in central China, with the largest number of malaria cases reported during the years 2005–2010. Different parasite genotypes are circulating in each endemic area of malaria-hypoendemic regions of Southeast Asia, and that geographic isolation may exist [7]. Additionally, with increasing movement of human populations, the *P. vivax* populations and transmission pattern in the regions are also changing constantly [20]. However, the genetic diversity of *P. vivax* parasites circulating in the area of central China is poorly understood. To better understand the *P. vivax* populations and transmission dynamics in Anhui of central China, the extent of genetic diversity of *P. vivax* parasites circulating in Bozhou of Anhui province were investigated using three polymorphic genetic marker *pvmsp-3α*, *pvmsp-1*, and *pvcsp* genes, and their relationships with parasites from other regions of China and Asian countries were analysed in this study. The data in the present study indicate that there was some degree of genetic diversity of *P. vivax* in Anhui province, and there appeared to be signs of genetic exchange and genetic recombination among vivax parasites in the region. Although a relatively small number of samples (45) could be obtained, the patients came from many parts of the city. Additionally, the diverse genetic backgrounds from the three genetic markers suggested that the parasites were not from outbreaks of 1–2 parasite strains and should be able to represent the genetic backgrounds of the parasite populations in the region.

It has been proposed that *pvmsp-3α* is a reliable molecular marker for analysis of *P. vivax* populations [11]. Based on the length of PCR products, four different allelic types of *pvmsp-3α* have been characterized around the world: type A (~1.9 Kb), type B (~1.5 Kb), type C (~1.1 Kb), and type D (~0.3 Kb) [6,21]. In the present study, only type A (~1.9 Kb) was identified among the 45 Anhui isolates tested. The observation was different from a previous report from several locations in China [7], in which type A, B, and C were detected in *P. vivax* isolates from Suzhou city of Anhui province, Sanya city of Hainan province, and Dehong prefecture of Yunnan province. The lack of other *pvmsp-3α* allele types in Bozhou city of Anhui province could simply be due to the relatively small sample size of this study. The results in some degree are consistent with the reports of type A being the most prevalent type around the world, with a frequency 70% to 100% (average ~80%) in many regions of China and countries in Asia and South America [7,21-27]. Seven patterns were detected in the *pvmsp-3α* gene after digestion of the PCR products with *HhaI*, with the most frequent allele variant being PH1 subtype. The results were consistent with those reported from other regions of the world, including those from Myanmar (n = 14) [7], Iran (n = 6) [14], Colombia (n = 9) [22], French Guiana (n = 11) [23], Thailand (n = 6) [28], Venezuela (n = 9) [29], and Peru (n = 17) [30]. However, outside Anhui province, the allele PH3, the second most common one in the present study, was the most prevalent in other areas of China including Hainan and Yunnan provinces, and also in Myanmar [7]. All of the *Hha*I allelic types found in the present study have been reported in other regions of the world, including Myanmar (PH1, PH3, PH4, PH5, and PH6) [7], India Chennai (PH1, PH4, PH5, and PH6) [11,21], Colombia (PH1, PH2, PH3, PH4, PH5, and PH6) [22], Brazil (PH3 and PH4) [26], and Thailand (PH1, PH3, PH4, PH6, and PH7) [28]. In China, five of the seven alleles were reported previously, e.g., PH1, PH3, PH4, PH5, and PH6 from Suzhou city of Anhui province; PH3, PH4, PH5, and PH6 from Hainan province; and PH1, PH3, and PH4 from Yunnan province [7].

Six patterns were detected in the *pvmsp-3α* gene after digestion of the PCR products with *AluI* in the present study, with the most predominated PA1 pattern in Bozhou city. This observation has not been reported in other areas of the world. Some allele types of *Alu*I digestion were comparable to those reported in other areas of the world, such as Colombia (PA2, PA4, PA5, and PA6), India (PA1, PA2, PA4, PA5, and PA6), and Iran (PA1 and PA2), suggesting that these allele types of *P. vivax* may have a global distribution [14,21,22]. The allele variant PA3 was a new allele identified in this study. The analyses from *pvmsp-3α* suggest that *P. vivax* populations in Bozhou city shared the majority of allelic variants with other parts of China and the world; however, some new allelic types were emerging.

To obtain more information of genetic polymorphism of the vivax malaria samples, the DNA sequences encoding the *pvmsp-1* and *pvcsp* genes were analysed. Three allele-types, Belem, Sal-I, and recombination types of the variable block 5 have been described from *P. vivax* isolates worldwide [8,16]. Genetic studies of *P. vivax* malaria parasites using the *pvmsp-1* marker have been reported from many countries, including India, Myanmar, Pakistan, and Korea [15,25,31,32]. In the present study, only Sal-I and R-types were detected among the Anhui isolates with R-type being the predominant allele types (75.6%). It has been reported that the R-type result from intragenic recombination of Belem and Sal-I types in mosquito vector [33,34]. The results indicate that inter-allelic recombination can be an important source of *pvmsp-1* genetic diversity among Anhui province locations. The high level of inter-allelic recombinant types in this isolate was in concordance with previous reports in Pakistan [25], India [32], Colombia [32], Thailand [35], and Brazil [36]. Although the Belem type is the predominant allelic type in other *P. vivax* malaria endemic regions in the world, including northern Iran [14], south-eastern and south-western parts of Afghanistan [37], and Azerbaijan [38], Belem type was not found among the Anhui isolates in this study. The absence of Belem type is intriguing, considering the presence of the recombinant types, which could be related to population changes of mosquito species or sampling bias. It has been reported that the distributions of different *P. vivax* populations in southern Mexico were largely determined by their infectivity to two species of anopheline vectors in the areas [39]. A similar situation could occur in China too; changes in mosquito strains or species could favor a subpopulation of vivax parasites, leading to changes in parasite population structure.

For *pvcsp* gene, three sequence types of VK210, VK247, and *P. vivax*-like have been detected based on a central repeat domain that varies in sequences and number of repeat units [17-19,40,41]. VK210 being the predominant type has been observed in Myanmar (98.3%) [15], Iran (69.3%) [24,42], Thailand (77%) [28], Afghanistan (86.6%) [37], Pakistan (95.7%) [37], Azerbaijan (100%) [38], Brazil (86%) [40], Guyana (92%) [41], and India (99.3%) [43], although VK247 was also reported to be the predominant type previously [44-46]. The VK247 and *P. vivax*-like types were not detected in the Anhui isolates in the present study. It has been proposed that the geographical differences in distribution of different parasite types may be related to the susceptibility of different species of *Anopheline* vectors to the infections by different malaria parasites in endemic areas [47,48]. The main malaria vectors in Anhui province are *An. sinensis* and *An. anthropophagus*, but the density of *An. anthropophagus* is very low in recent years, and the re-emergence of *P. vivax* malaria was mainly caused by *An. sinensis*[49]. Therefore, the prevalence of VK210 type may be correlated with the increase of *An. sinensis* malaria vector in the region. A total of 12 VK210-1 subtypes were identified in 45 blood samples from Anhui province, two subtypes of which, VK210-6 and VK210-10, were also detected in other endemic areas, including China’s Tibet, North Korea, and South Korea through phylogenetic and Blast analysis.

In summary, *P. vivax* populations in Bozhou city of Anhui province have some degree of genetic diversity in three highly polymorphic markers, with 16 unique haplotypes observed among 45 samples (~36%). However, the present results also showed that *P. vivax* populations in Bozhou city of Anhui province had relative even major genotypes, *pvmsp-3α* type A (100%), *pvmsp-1* R2 (75.6%), and *pvcsp* VK210 subtype (100%), suggesting its relative uniform parasite populations. It is interesting that *pvmsp-1* recombinant type was detected, which may be related to the recent changes in vector population (increase in *An. sinensis* mosquito) in the region, or may have been simply sampled some subpopulations from recent outbreaks. The data in this study showed shared genotypes with those from Korea and other countries; there is a possibility that these parasites have the same origin and were disseminated by travelers. Although limited samples were included in this study, and the data may not represent the whole picture of genome diversity of the parasite populations in the region, the results suggest parasite populations with relatively diverse genetic backgrounds in the region. The genetic diversity information obtained may provide valuable information for tracking and monitoring future malaria infections in this region.

## Competing interests

The authors declare that they have no competing interests.

## Authors’ contributions

XZS and FL designed and supervised the study, analysed the data, and wrote and revised the manuscript. BH and SH carried out the experiments, and analysed the data, and wrote the manuscript. HG, YX, FX, XH, YY, and SW participated in field work and preliminary data analysis. All authors read and approved the final manuscript.

## Supplementary Material

Additional file 1 **Sequences of the primers and cycling conditions used to amplify the *pvmsp-3α*, *pvmsp-1*, and *pvcsp *** **genes of *P. vivax *** **isolates from Anhui province.** The data provided oligonucleotide primers and cycling conditions of *pvmsp-3α*, *pvmsp-1*, and *pvcsp* genes. Click here for file
