# Supplementary material for: Genetic diversity of Plasmodium vivax population in Anhui province of China
Source: Malar J. 2014 Jan 8;13:13. doi: 10.1186/1475-2875-13-13 (PMC3893497; doi:10.1186/1475-2875-13-13)
Supplement: Additional file 1 — Sequences of the primers and cycling conditions used to amplify the pvmsp-3α , pvmsp-1 , and pvcsp genes of P. vivax isolates from Anhui province. The data provided oligonucleotide primers and cycling conditions of pvmsp-3α, pvmsp-1, and pvcsp genes. [file 1475-2875-13-13-S1.doc]

**Additional file 1**

Sequences of the primers and cycling conditions used to amplify the *pvmsp-3α*, *pvmsp-1*, and *pvcsp* genes of *P. vivax* isolates from Anhui province.

| Genes^△^ | Primers* | PCR cycling conditions^▲^ | Product size (bp) | References |
| --- | --- | --- | --- | --- |
| *pvmsp-3α* (P) | F: 5'-CAGCAGACACCATTTAAGG-3' | 94°C 3 min/[94°C 30 s, 56°C 30 s, 68°C 2.5 min] × 35 cycles, 68°C 7 min | 2195 | [10] |
|  | R: 5'-CCGTTTGTTGATTAGTTGC-3' |  |  |  |
| *pvmsp-3α* (S) | F: 5'-GACCAGTGTGATACCATTAACC-3' | 94°C 3 min/[94°C 30 s, 57°C 30 s, 68°C 2.5 min] × 35 cycles, 68°C 7 min | 1896 | [10] |
|  | R: 5'-ATACTGGTTCTTCGTCTTCAGG-3' |  |  |  |
| *pvmsp-1* (P) | F: 5'-GATGGAAAGCAACCGAAGAAGGGAAT-3' | 95°C 6 min/[95°C 40 s, 50°C 60 s, 72°C 1.5 min] × 30 cycles, 72°C 7 min | 1784 | [13] |
|  | R: 5'-AGCTTGTACTTTCCATAGTGGTCCAG-3' |  |  |  |
| *pvmsp-1* (S) | F: 5'-ACTACTTGATGGTCCTC-3' | 94°C 5 min/[94°C 40 s, 58°C 60 s, 72°C 60 s] × 35 cycles, 72°C 7 min | 1454 | [14] |
|  | R: 5'-TTGTGACATGCGTAAGCG-3' |  |  |  |
| *pvcsp* (P) | F: 5'-ATGTAGATCTGTCCAAGGCCATAAA-3' | 95°C 6 min/[95°C 40 s, 58°C 60 s, 72°C 60 s] × 25 cycles, 72°C 7 min | 1006 | [13] |
|  | R: 5'-TAATTGAATAATGCTAGGACTAACAATATG-3' |  |  |  |
| *pvcsp* (S) | F: 5'-GCAGAACCAAAAAATCCACGTGAAAATAAG-3' | 95°C 6 min/[95°C 40 s, 62°C 60 s, 72°C 60 s] × 35 cycles, 72°C 7 min | 683 | [13] |
|  | R: 5'-CCAACGGTAGCTCTAACTTTATCTAGGTAT-3' |  |  |  |

^△^**P**: Primary PCR reaction; **S**; Secondary PCR reaction.***F**; Forward primer; **R**; Reverse primer. ^▲^The cycling conditions were modified in the present study.
